# Acupuncture treatment of hypertension with insomnia: A protocol for randomized, double-blind, placebo controlled trial research

**DOI:** 10.1097/MD.0000000000028455

**Published:** 2022-01-14

**Authors:** Xun Zhu, Luda Yan, Xuejiao Dou, Yanping Zheng, Guanglin He, Meiyan Liao, Wenbin Fu

**Affiliations:** a The Second Clinical College, Guangzhou University of Chinese Medicine, Guangzhou, P.R China; b Department of General Practice, Bao’an People's Hospital, Shenzhen, P.R. China; c Department of Acupuncture and Moxibustion, Bao’an Traditional Chinese Medicine Hospital, Shenzhen, P.R. China; d Department of General Practice, Bao’an Traditional Chinese Medicine Hospital, Shenzhen, P.R. China; e Department of Acupuncture and Moxibustion, The Second Affiliated Hospital of Guangzhou University of Chinese Medicine (Guangdong Provincial Hospital of Chinese Medicine), Guangzhou, P.R. China; f Innovative Research Team of Acupuncture for Depression and Related Disorders, The Second Affiliated Hospital of Guangzhou University of Chinese Medicine (Guangdong Provincial Hospital of Chinese Medicine), Guangzhou, P.R. China.

**Keywords:** acupuncture, hypertension, insomnia, RCT

## Abstract

**Introduction:**

Hypertension patients often suffered from insomnia problems which lowered the quality of life. Studies have shown that acupuncture is effective to treat perimenopausal and cancer-related insomnia. However, there is a lack of randomized controlled trials to support the effectiveness of acupuncture on insomnia of hypertension patients.

**Methods and analysis:**

This study is a randomized, double-blind (patients and evaluators), and placebo-controlled clinical trial to investigate the effect of acupuncture in hypertension patients’ insomnia management. We will recruit 158 hypertension patients suffering from insomnia in Bao’an People's Hospital, Shenzhen and randomly assign them into treatment group (antihypertensive drugs + acupuncture) and control group (antihypertensive drugs + sham acupuncture) in a 1:1 ratio. The patients will receive acupuncture 3 times a week for 12 weeks, and then a 6-months follow-up will be conducted after the treatment. The primary outcome is the Pittsburgh Sleep Quality Index. The secondary outcomes include sleep parameters, blood pressure dropping, sleeping pill dosage, Rating Depression Scale score, and Self-Rating Anxiety Scale score. The primary outcome will be evaluated at baseline, 4, 8, and 12 weeks, and 1, 3, and 6 months following the end of treatment. The secondary outcomes will be assessed at baseline and 12 weeks of the treatment period.

## 1 Introduction

Hypertension is a common chronic disease, and it is estimated that 972 million (26.4%) adult hypertension patients in 2000 will increase to 1.56 billion (29.2%) in 2025 worldwide.^[1]^ Hypertension often causes dizziness and headache, leading to several insomnia problems such as difficulty in falling asleep, easily waking up at night, or low sleep efficiency.^[2]^ Insomnia reduces productivity, lowers life quality, and impairs cognitive and emotional functions.^[3]^ If without proper treatment, insomnia can be developed as a chronic disease and increases the incidence of hypertension.^[4]^ Therefore, it is important to explore the adequate therapy to improve the insomnia of hypertension patients.

Currently, drug therapy and psychotherapy are the main methods to treat insomnia; however, psychotherapy exists some problems, such as the shortage of therapists and heavy economic burden for patients. Simultaneously, most drugs mainly effect on anti-anxiety, sedation and hypnosis, and long-term use may produce adverse reactions and drug dependence.^[5]^ In addition, it is easy to relapse or appear withdrawal symptoms after drug stopping.^[6]^ Acupuncture is an important part of traditional Chinese medicine, and has been widely used in China and some western countries. Presently, more and more attention has been paid to the clinical effect of acupuncture on insomnia. Study has showed that acupuncture increases the percentage of slow-wave sleep to improve the sleep quality.^[7]^ Fu et al^[8]^ have studied the effectiveness of acupuncture on perimenopausal insomnia, and found that acupuncture increased sleep efficiency and sleep time, decreased wakefulness, and improved sleep structure, showing that acupuncture could improve the insomnia from both subjective and objective aspects. Yin et al^[9]^ also found a good effect of acupuncture on the improvement of primary insomnia. To our best knowledge, whether acupuncture could be effective on the insomnia of hypertension patients has not been reported.

Given the lack of studies on acupuncture in hypertension patients’ insomnia management, we conducted a randomized, double-blind, and placebo-controlled trial so as to evaluate the efficacy and safety.

## 2 Methods and analysis

### 2.1 Study design

**Figure 1:**
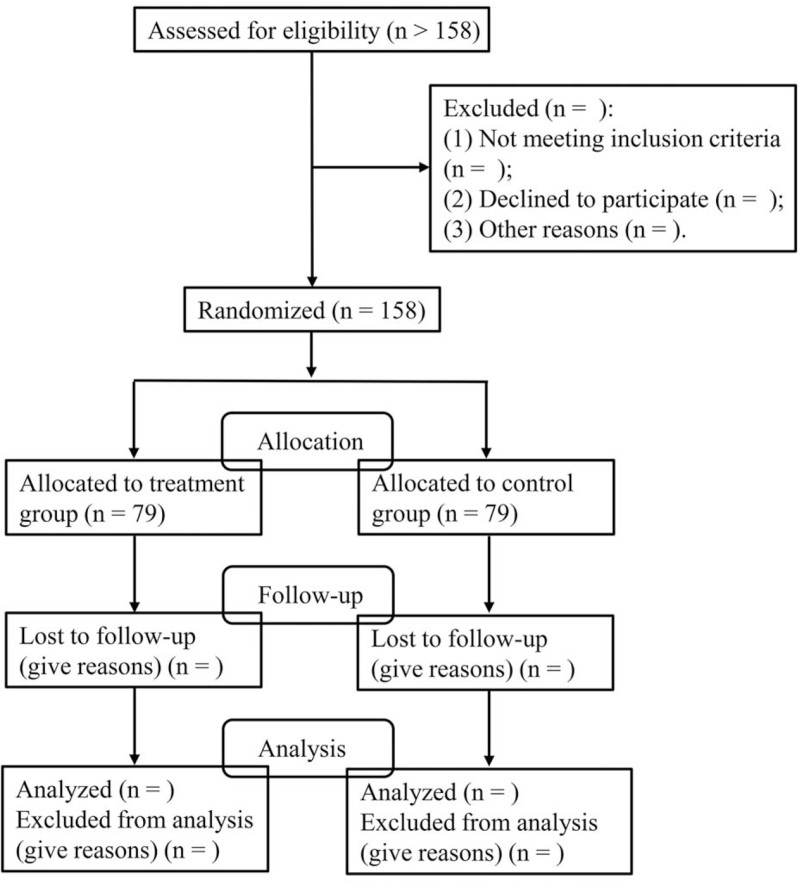
The flow chart of trial process.

This study is a randomized, double-blind, and placebo-controlled trial to evaluate the efficacy and safety of acupuncture on hypertension patients’ insomnia management, and the flow chart of this trial is displayed in Figure 1. The trial will be performed in Bao’an People's Hospital, Shenzhen. The study has been approved by the Ethics Committee of Bao’an People's Hospital, Shenzhen (BYL20210904), and registered on www.chictr.org.cn (ChiCTR2100051063).

### 2.2 Patients

The hypertension patients with insomnia will be recruited in Bao’an People's Hospital, Shenzhen from September, 2021 to March, 2022. Each patient uses 1 to 3 antihypertensive drugs (calcium antagonist, angiotensin converting enzyme inhibitor/angiotensin receptor blocker, diuretics, or β-blocker). On the basis that patient's diet, physical activity, and use of antihypertensive drugs remain unchanged, they will accept a 12-week treatment. Each patient is required to sign the informed consent before the treatment, and accept a 6-month follow-up (visits at 1, 3, 6 months).

### 2.3 Inclusion criteria

The patients should meet all the following criteria:

- age between 18 and 75 years;
- meeting the diagnostic criteria of hypertension (systolic blood pressure 140-180 mm Hg and/or diastolic blood pressure >90 mm Hg);
- insomnia diagnosed in accordance with the International Classification of Sleep Disorders^[10]^;
- Pittsburgh Sleep Quality Index (PSQI) score >5;
- not receiving acupuncture for at least 1 year;
- agreeing to participate in this clinical study and signed the informed consent.

### 2.4 Exclusion criteria

The patients will be excluded if they meet 1 of the following criteria:

- with secondary hypertension;
- with severe complications, such as cardiovascular events, endocrine disorders, renal, and hematopoietic system disease;
- with comorbidities including cancer and mental illness;
- pregnant or lactating women;
- having skin infections near the specified acupoints;
- with insomnia caused by external environment disturbances;
- with severe chronic obstructive pulmonary disease and bronchial asthma;
- with severe neurological dysfunction;
- with alcohol abuse or drug dependence;
- who considered by the researcher to be unsuitable for this study.

### 2.5 Randomization and blinding

Both patients and evaluators will be blinded to the allocation. After signing the informed consent, the eligible patients will be randomly divided into treatment group (acupuncture group) and control group (sham acupuncture group) at 1:1 ratio. The random coding table (001-158) was used for the allocation, and serial numbers was in accordance to the number of patients. The random coding table will be preserved and managed by the designated personnel. After the patients included in this study, their number is notified by researchers to the designated personnel, who will instruct the patients into treatment group or control group. The researchers will write down corresponding records and follow the allocation.

### 2.6 Treatment group

Patients in the treatment group will accept acupuncture for 12 weeks, with 3 times a week. Baihui (GV20), Yintang (GV29), Renying (ST9), Hegu (LI04), and Taichong (LR3) will be used as the main acupoints. Acupuncturists will be allowed to add acupoints based on traditional Chinese medicine syndrome differentiation and treatment. The needles will be inserted into the skin to a depth of 10 to 30 mm, and manually operated (including lifting, inserting, and rotating) until patients report a tingling sensation (Deqi sensation). The needles on GV20 and GV29 will be connected to the G6805-2 Multi-purpose Health Device (Huayi Company, Shanghai, China) with frequency at 2.5 Hz and intensity of 45 mA using continuous wave type. The needles will be kept for half an hour before removal.

### 2.7 Control group

Patients in the control group will accept sham acupuncture. A non-invasive placebo control (the Streitberger Placebo-needle) will be used. The acupoints are the same as the treatment group, with no insertion. The electroacupuncture instrument (G6805-2 Multi-purpose Health Device) will be placed beside the patients and connected to GV20 and GV29, without electric pulse. Before removal, the needles will be also kept for 30 minutes.

### 2.8 Outcome measurement

#### 2.8.1 Primary outcome measurement

The primary outcome will be assessed using PSQI, a self-rating questionnaire designed to assess overall sleep quality. It includes 19 self-rating items and 5 other-rating items. It evaluates sleep quality according to the following 7 aspects over the past month: sleep duration, sleep efficiency, daytime dysfunction, delays in falling asleep, use of medications to sleep, sleep disorders, and overall sleep quality. Each aspect is rated 0 to 3 points, and the accumulated scores of the 7 aspects are 0 to 21 points. The higher score indicates poorer sleep quality. The reliability and validity of the Chinese version have been verified.^[11]^ The PSQI will be evaluated at baseline, during treatment period (at 4, 8, and 12 weeks) and follow-up period (at 1, 3, and 6 months).

#### 2.8.2 Secondary outcome measurement

The secondary outcomes are sleep parameters, blood pressure dropping, sleeping pill dosage, Rating Depression Scale (SDS) score, and Self-Rating Anxiety Scale (SAS) score.

The sleep parameters, including sleep efficiency, total sleep time (TST), sleep arousal, arousal index, wakefulness after sleep onset, are obtained by Insomnia Severity Index (ISI) scores and nocturnal polysomnography. ISI^[12]^ is designed to evaluate the insomnia symptoms at day and night. The questionnaire consists of 7 items, and each item scores from 0 to 4, and the total score ranges from 0 to 28. The higher score indicates the worse insomnia. The insomnia is classified to be clinical (0-7 points), mild (8-14 points), moderate (15-21 points), and severe (22-28 points). ISI is a subjective self-report questionnaire, whereas nocturnal polysomnography includes many quantitative parameters (arousal index, wakefulness after sleep onset, TST, etc). The patients’ wrist will be worn with the wActiSleep-BT actigraphy (Actigraph LLC, Pensacola, FL) to record sleep quality by noting sleep arousal, TST, sleep onset time, sleep latency, and sleep efficiency. The ActiLife6 (Actigraph LLC, Pensacola, FL, USA) software is used to analyze sleep status and sleep quality.

Patients will be allowed to take sleeping pill (estazolam, 0.5-2.0 mg) during the trial if have a difficulty in sleeping for 3 consecutive days. They will be asked to write down the dose and time of estazolam on a case report form (CRF).

SAS^[13]^ and SDS^[14]^ are used to assess anxiety and depression. The SAS questionnaire consists of 20 items, including 15 negative and 5 positive descriptions. The total score >40 indicates a state of anxiety, which is more severe with the higher score. The SDS also consists of 20 items, and the standard score is calculated by sorting patients’ answers to the 20 questions, with a total score of 1.25 times the standard score. The cutoff point for depression was 50, and the higher score indicates the higher degree of depression.

### 2.9 Data collection and management

Evaluation will be conducted during the screening and preparation phases and then periodically conducted throughout the trial. The screening phase will last for 2 weeks, and general information includes age, sex, height, weight, previous medical history, blood pressure, and medication history. The data collection and time course are listed in Table 1.

**Table 1:** Time course for data collection and follow-up.

Enrollment and allocation	Enrollment and allocation		Treatment period			Follow-up period		
Time point	Screening (−14∼0 d)	Allocation (0 d)	4 wk	8 wk	12 wk	1 mo	3 mo	6 mo
Demographics	X							
Vital signs	X							
Previous medical history	X							
Inclusion/exclusion assessment	X							
Informed consent		X						
Routine blood	X							
Routine urine test	X							
Liver function	X							
Kidney function	X							
Electrocardiograph	X							
Allocation		X						
Treatment		X	X	X	X			
Primary outcome		X	X	X	X	X	X	X
Secondary outcomes		X			X			
Adverse events		X	X	X	X	X	X	X

The primary data of patients are collected, and then transcribed into the CRF by the researcher. The researcher must completely and truly record the data according to the clinical study protocol. Data significantly deviating from the clinically acceptable range must be checked and explained. The completed and verified CRF will be handed over to the relevant personnel for data entry, management and statistics. After the transference, the data on the CRF will not be modified. Results will be personally explained to each participant, and disseminated as articles published in international peer-reviewed journals. To publish, we will adhere to the official eligibility guidelines for authorship, and do not tend to use professional writers.

### 2.10 Patients’ safety

All patients will accept blood routine, hepatic, and renal functions tests to identify and exclude the patients with severe heart, liver, or kidney disease after the recruitment and before the random allocation. The acupuncture will be discontinued if patients occur any adverse effects. Adverse events (AE) indicate any adverse medical events that occur in the patients regardless of events are related to the operation or experimental drug in the study. The patients will be informed to contact the doctor to diagnose and treat the AE. Fainting, allergy, and pain may appear in the acupuncture clinical trials.^[15]^ If faints occur, the needles will be pulled out immediately, patients will be returned to supine position, given warm or sugar water, and rest until complete recovery. If allergy or pain occurs, needles are taken out immediately and patients are treated according to the specific situation.

The serious AE (SAE) includes death, life-threatening conditions, severe or persistent incapacity or disability, hospitalization, extension of hospitalization time, and potentially serious events. Whether relating to the trial, any SAE or relevant events must be reported to the research unit within 24 hours. The researcher is responsible for recording and reporting all AE and SAE that occurred during the trial (including a 12-week treatment period and a 6-month follow-up period) since the date of signing the informed consent.

### 2.11 Withdrawal criteria

The patients presenting the following conditions will be withdrawn from this study:

- occurring serious adverse reactions so as to be inappropriate to continue the study;
- occurring severe complications or condition deterioration so as to need emergency measures;
- not following the prescribed treatment or without complete data so as to affect the efficiency of evaluation.

### 2.12 Sample size

PSQI scores after the end of acupuncture are taken as the sample size calculation index. The statistical significance level is set as 0.05, and 80% is taken as the statistical power. A previous study showed the PSQI score of patients with perimenopausal insomnia is 8.62 ± 2.93 in the acupuncture group and 14.76 ± 3.35 in the sham acupuncture group.^[8]^ According to this, the following formula is used to calculate the estimated sample size: N = 2[(t_α/2_ + t_β_)S/δ]^2^. The combined standard deviation is assumed as 1.5, and δ is 0.5 times of the standard deviation. β is 0.8, and α is 0.05. Considering the dropout rate of 20%, at least 79 cases will be required in each group. Therefore, we need to recruit a total of 158 patients for this randomized controlled trials (RCT).

### 2.13 Statistical analysis

The statistical analysts will be blind to the patients’ group allocation. All data, including data of the withdrawing patients during the trial, will be analyzed based on intention-to-treat. Two different researchers will participate in the data entry, and all data are entered twice to guarantee the accuracy. The statistical analysis will be performed using SPSS 24.0 software (IBM, Armonk, NY, USA). The measurement data will be expressed as mean ± standard deviation or median and separately compared using *t* test and Wilcoxon rank-sum test. The count data will be shown as number and percentage, and compared using chi-squared or Fisher exact test. The change of PSQI score between the 2 groups at different time points will be compared by repeated-measures analyses of variance. The difference before and after treatment in each group will be determined using paired *t* test. All statistical tests are 2-tailed, with *P* values ≤.05 as statistical significance.

## 3 Discussion

Hypertension patients usually suffer from insomnia, leading to a poor quality of life.^[2,3]^ Moreover, insomnia further increases the risk of cardiovascular disease, especially hypertension.^[4]^ To improve the insomnia of hypertension patients, the proper treatment needs to explore. Acupuncture has been practiced in China more than 2000 years and has become widely applied in western countries in recent years.^[16,17]^ The current studies have showed that acupuncture can be used to relieve primary, perimenopausal, and cancer-related insomnia symptoms,^[18–20]^ but the effect on the insomnia of hypertension patients has not been determined. This randomized, double-blind and placebo-controlled trial aims to assess the effect of acupuncture in hypertension patients suffering from insomnia, which may provide high-quality clinical evidence for the treatment of acupuncture in the insomnia of hypertension patients.

Insomnia is mainly a subjective feeling, and the PSQI and the ISI used to evaluate sleep quality and insomnia severity are both scored based on patients’ subjectivity.^[11,12]^ The SAS and SDA questionnaires used to evaluate anxiety and depression are still subjective scores from insomnia patients and exist uncertainties.^[13,14]^ The controversies existing in the study on the efficacy of acupuncture are mainly based on the following points. First, either researcher or patient will appear psychological effect (placebo effect), which may cause deviation to the results. Second, the efficacy of acupuncture does not always depend on the specific acupoints, and the sham acupuncture intervention are suitable for the acupoints unrelated to sleep physiology.^[21]^ A systematic review from Yang et al^[22]^ reported that acupuncture showed a greater short-term hypotensive effect than non-sham acupuncture in controlled trials, which probably reflected a bias that the acupuncture showed no true hypotensive effect. Therefore, Yang et al^[22]^ recommended that future RCTs must adopted sham acupuncture as control to decrease the bias. Our study uses a strict RCT with double-blind method and sham acupuncture as control, which may decrease the bias in a larger extent and reflect the true effect of acupuncture. In addition, the wrist-worn sleep monitor (actigraphy) is applied to detect the sleep quality, which can objectively and accurately evaluate the therapeutic effect of acupuncture.

Our protocol provides a standardized process and we will follow each step of this protocol to conduct the subsequent clinical study. We expect that this trial will generate strong clinical evidence for the efficiency of acupuncture in hypertension patients’ insomnia management.

## Author contributions

**Conceptualization:** Xun Zhu, Wenbin Fu.

**Data curation:** Luda Yan, Xuejiao Dou, Yanping Zheng, Guanglin He, Meiyan Liao.

**Formal analysis:** Luda Yan, Xuejiao Dou, Yanping Zheng, Guanglin He, Meiyan Liao.

**Investigation:** Luda Yan, Xuejiao Dou, Yanping Zheng, Guanglin He, Meiyan Liao.

**Methodology:** Luda Yan, Xuejiao Dou, Yanping Zheng, Guanglin He.

**Writing – original draft:** Xun Zhu.

**Writing – review & editing:** Xun Zhu, Wenbin Fu.
